# Clinical perspectives on the association between respiratory syncytial virus and reactive airway disease

**DOI:** 10.1186/rr186

**Published:** 2002-06-24

**Authors:** Nele Sigurs

**Affiliations:** 1 Department of Pediatrics, Borås Central Hospital, Borås, Sweden

**Keywords:** allergy, asthma, bronchiolitis, reactive airway disease, respiratory syncytial virus

## Abstract

Asthma is a leading cause of morbidity and mortality among children worldwide, as is respiratory syncytial virus (RSV). This report reviews controlled retrospective and prospective studies conducted to investigate whether there is an association between RSV bronchiolitis in infancy and subsequent development of reactive airway disease or allergic sensitization. Findings indicate that such a link to bronchial obstructive symptoms does exist and is strongest for children who experienced severe RSV illness that requires hospitalization. However, it is not yet clear what roles genetic predisposition and environmental or other risk factors may play in the interaction between RSV bronchiolitis and reactive airway disease or allergic sensitization. Randomized, prospective studies utilizing an intervention against RSV, such as a passive immunoprophylactic agent, may determine whether preventing RSV bronchiolitis reduces the incidence of asthma.

## Introduction

Childhood asthma is a serious global public health problem. According to the World Health Organization [1], asthma is the most common chronic disease in children. In some areas of the world the incidence in children is over 50% [1]. Although no single specific cause of asthma has been found, a number of predisposing factors have been identified and are the targets of ongoing clinical investigations. Lower respiratory tract infection (LRTI) caused by respiratory syncytial virus (RSV) in infancy has been identified as one such potential risk factor.

Nearly all children are infected with RSV and develop IgG antibodies to RSV during the first 2 years of life [2]. Although infection is often mild and limited to the upper respiratory tract, RSV is the leading cause of LRTI worldwide [2]. As many as 40% of infants may develop bronchiolitis or pneumonia, and up to 2% of all infected children are hospitalized for severe RSV disease every year [3]. Children who are born premature or who have chronic illnesses are at even greater risk for serious infection and hospitalization from RSV [3].

RSV bronchiolitis is characterized by expiratory wheezing and respiratory distress [4]. Although multiple studies [4-17] have reported that infants with RSV bronchiolitis often develop recurrent wheezy bronchitis and asthma during childhood, several questions regarding the link between RSV and reactive airway disease (RAD) must be addressed. First, is there a direct connection between severe and/or mild RSV infection and subsequent wheezing/asthma, or are there other factors – perhaps hereditary or environmental – that might account for a predisposition to both RSV and RAD? Second, is the severity of infection predictive of sequelae? In other words, is there a difference in the risk for subsequent bronchial obstructive symptoms between children with RSV infection that requires hospitalization and those with milder infections? Finally, is there a connection between RSV and subsequent allergic sensitization?

This report reviews the available literature that addresses the potential links between RSV and RAD and between RSV and allergy/atopy. Both controlled retrospective and prospective studies are evaluated.

## Relationship between RSV bronchiolitis in hospitalized children and subsequent RAD: controlled studies

Table 1 provides a summary of available follow-up studies with control groups in both hospitalized and non-hospitalized children.

Controlled retrospective studies of infants hospitalized for RSV LRTI date back several decades. In 1978, Sims *et al*. [5] assessed 8-year-old children (*n* = 35) who had RSV bronchiolitis in infancy and 35 control children matched for age, sex, and socioeconomic status [5]. A clinical history gathered from interviews with parents ascertained any evidence of wheezing in the children or close relatives, as well as any allergic rhinitis, urticaria, or smoking among family members. A physical examination was performed, including standard respiratory function tests (forced expiratory volume at 0.75 s [FEV_0.75_], vital capacity, peak expiratory flow rate [PEFR] at rest, and FEV_0.75_ as a percentage of vital capacity). In these children, the mean exercise bronchial lability of the index children was significantly greater than that in control children (*P* < 0.01) and the mean PEFR at rest was significantly lower (*P* < 0.02), although Sims *et al*. suggested that environmental factors such as smoking might also play a role.

A subsequent study conducted in 1982 by Pullan and Hey [6] confirmed those findings, and hypothesized that some children might be more susceptible to severe infection because of pre-existing changes in their airways. These changes could be due to impaired defense mechanisms or environmental factors, such as second-hand smoke, family size, or crowded sleeping arrangements. Those investigators reviewed the records of 180 children admitted during their first year of life with confirmed RSV infection. They were able to interview and examine 130 of the children 10 years later, with a full pulmonary work-up in 107 children; 111 control children were also included. Wheezing following LRTI was noted in 42% of the index children and in 19% of control children (*P* < 0.001); an increase in the incidence of bronchial lability was also noted in the index children. Pullan and Hey felt that the increased bronchial lability may have been related to RSV infection during infancy, although the link to asthma was unclear. Most excess wheezing occurred during the first 4 years of life, but was present in some children up to age 10 years. Indicators of atopy (e.g. eczema, rhinitis, and positive skin tests) were not found to be more common in index than in control children. There was no between group difference in family history of atopy.

In 1982 and 1984, Mok and Simpson [7,8] reported their findings from a retrospective study of 200 children who had been hospitalized for LRTI in infancy; 100 of these children had confirmed RSV disease. Those investigators compared historical data and ventilatory function in these children with those of 200 control children at 7 years after LRTI. Bronchitis and asthma were more common and more severe in the RSV group. All of the 200 index children had bronchial hyperreactivity and abnormal pulmonary function at follow up, regardless of the type of LRTI they had been diagnosed with initially. Again, as with previous studies, no relation with family or personal history of atopy was identified [8].

Three further studies [9-11] examined whether acute bronchiolitis was a predictor of reversible obstructive airway disease. In the earliest of those studies [9], 51 infants hospitalized for acute bronchiolitis over a 2-year period were included and were examined every 6 months up to age 2 years. These children were matched with 24 control children. At the first episode of bronchiolitis and at any time the children were diagnosed with bronchopulmonary obstruction, nasopharyngeal swabs were collected for virological examination; acute and convalescent sera were collected to check viral antibody titers. Skin prick tests were conducted to ascertain allergic status. Blood tests for IgE, IgG, IgM, IgA, and eosinophil counts were taken at hospital admission and at age 2 years in index children, and at age 2 years in control children. Children who had been hospitalized for acute bronchiolitis had twice as many respiratory infections as did control children (*P* < 0.01). By age 2 years, 60% of these children had three or more episodes of bronchopulmonary obstruction, as compared with only one child who had symptoms in the control group. RSV was identified in 61% children during their initial bout of bronchiolitis. The authors felt that the viral infection may have damaged the respiratory tract and increased the likelihood of subsequent severe illness in these children. They also felt that it was possible that a structural defect or immaturity of the airways (in preterm infants) was a predisposing factor for bronchiolitis. Those investigators were not able to demonstrate any factor (including atopy) that separated the index children from control children in terms of either susceptibility to acute illness or later symptoms.

In 1992, Murray *et al*. [10] reported on a cohort of 101 infants admitted to hospital for acute bronchiolitis over the course of three winter epidemics. Five and a half years after their initial admission, 73 of the children were re-evaluated and matched for age, sex, race, neighborhood, and history of maternal smoking with 73 control children. Of the original cohort 66% were RSV positive, whereas at the follow-up at age 6 years 68% were RSV positive. A further follow-up study [11] looked at some of these children (61 index cases and 47 control children) after 9.5 years. Index children at 9 or 10 years after hospitalization coughed more (48% versus 17%), wheezed more (34% versus 13%), and required more bronchodilators (33% versus 3%) than did control children. Asthma was diagnosed in 39% of index children as compared with 13% of control children based on those criteria. The results of lung function testing conducted to assess overall lung growth (i.e. forced vital capacity, functional residual capacity, total lung capacity) were not affected in these children. However, measures of lung function (PEFR, FEV_0.75_ and FEV_1.0_, and airway resistance measured plethysmographically) indicated the presence of airway obstruction significantly more commonly in the index children. Skin tests and family history failed to support atopy as an explanation for this inferior lung function or why these children were more likely to cough and wheeze. The study supported previous findings that family history of asthma did not predispose children to wheezing [7]. The only significant predictor of wheezing was a prior episode of bronchiolitis, often from RSV infection [10,11].

In 1993, Osundwa *et al*. [12] conducted a retrospective study of 70 children who had been hospitalized with RSV bronchiolitis, matched with 70 control children. All were reviewed 2 years later and it was discovered that nearly half (44% of index cases) developed recurrent wheezing, as compared with only 13% of control children (*P* = 0.001). Again, this finding was independent of any family history of atopy.

Weber *et al*. [13] conducted a prospective study that compared 105 children admitted for RSV LRTI (cohort group) with 105 children matched for age who were not admitted to hospital during the same RSV season and with another control group of 102 children born after the RSV season. All groups were followed up for the next few years. Both pneumonia and wheezing were more common in the cohort group than in both control groups during the first 2 years. By age 3 years, the rates had fallen to low levels in all groups and no significant differences were noted. Thus, for reasons not understood, the rate of postbronchiolitic wheezing in that study differs from the rates reported in other studies.

Sigurs and coworkers [4,14] conducted a prospective study to evaluate the association between the occurrence of bronchial obstructive symptoms and IgE antibodies after RSV bronchiolitis in infancy. The study population was a cohort of 47 previously healthy infants hospitalized for RSV bronchiolitis, who were matched for age, sex, and place of residence with a group of 93 control children. Children enrolled in the study had a mean age of 3.5 months at the start of the investigation. Children were examined at ages 1, 3, and 7.5 years for the presence of asthma and/or allergic sensitization. At age 7.5 years the cumulative prevalence of asthma was 30% in index cases who had had RSV versus 3% in the control group (*P* < 0.001). Current asthma was present in 23% of RSV-infected children as compared with 2% of the control group (*P* < 0.001). Children in the RSV infected group also had more current atopic asthma (i.e. asthma combined with specific IgE antibodies; 14.9% versus 1%; *P* = 0.002). Those children also had higher rates of allergic rhinoconjunctivitis (14.9% versus 2%; *P* = 0.007). Furthermore, the cumulative prevalence rates for any wheezing were 68% and 34% (*P* < 0.001), and the current prevalence rates for any wheezing at age 7.5 years were 38% and 2% (*P* < 0.0001) for cases and controls, respectively [14]. The RSV group and the control group were similar in terms of the frequency of atopic dermatitis. The two groups were compared extensively with regard to family history of atopy/asthma and other background factors, and no differences were found.

## Relationship between mild RSV bronchiolitis in infancy and subsequent RAD: controlled studies

Research conducted in children hospitalized for severe bronchiolitis or pneumonia secondary to RSV infection verified the link between this event in infancy and subsequent development of RAD [4-14]. However, does such an association exist for children who had milder RSV infections and were managed as outpatients?

McConnochie and Roghmann [15] compared 59 children at age 8 years who had been diagnosed with mild bronchiolitis of undetermined etiology during infancy. They matched 177 control children drawn from a normal pediatric population and found a highly significant difference in current wheezing (*P* < 0.0001), with 13.6% of control children versus 44.1% of index children experiencing these symptoms. When other variables (second-hand smoking, genetic predisposition, etc.) were analyzed, wheezing in 9.4% of the population of children was found to be attributable to bronchiolitis. Likewise, another study [16] reported the incidence of wheezing among a subpopulation of children who had been recruited from the German Multicenter Atopy Study. Those investigators found that the more obvious the asthma symptoms, the greater the prevalence of RSV seropositivity at age 1 year (*P* = 0.001).

The Tucson Children's Respiratory Study [17] enrolled a cohort of 1246 children in infancy and followed some of them prospectively to age 13 years. Of the 1246 children, 888 were followed for the first 3 years of life. A total of 519 children had at least one pediatrician-diagnosed LRTI during these 3 years. Of the 472 children who tested positive for viruses, approximately half (43.9%) were positive for RSV. Compared with the children in the cohort who had no LRTIs, children with even mild RSV LRTI had a significantly increased risk for frequent wheezing by age 6 years (*P* < 0.001). The RSV-positive group was 3.2 and 4.3 times more likely to have infrequent wheezing and frequent wheezing, respectively. The increased risk for wheezing remained statistically significant up to age 11 years but not to age 13. Again, the authors found no association between RSV infection and atopy.

## RSV infections and allergic sensitization

Although IgE antibodies normally comprise a small percentage of the total immunoglobulins in the body, they play an instrumental role in acute allergic reactions. IgE antibodies are the mediators of the type 1 hypersensitivity reaction. They cause degranulation of mast cells and release of histamine and other chemical mediators of the allergic response, which produce the characteristic wheal and flare on a positive skin prick test [18]. The relationship between RSV bronchiolitis and RAD is discussed above. Another important question is whether RSV and perhaps other viral respiratory tract infections play any role in subsequent development of allergy in children. The studies discussed below used serum IgE and/or skin prick tests to evaluate the relationship between RSV and allergic sensitization (Table 2).

### Studies of children with severe RSV infection

Welliver *et al*. [19] conducted a study of 78 infants who were hospitalized with RSV bronchiolitis at less than 6 months of age. Nasopharyngeal secretion samples were tested for the presence of RSV-specific IgE. Acute and convalescent specimens were obtained. Of those infants 38 were followed until age 4 years [19] and 43 were seen at age 7 or 8 years [20]. At age 7 or 8 years the children underwent skin prick testing, pulmonary function tests, and pulse oximetry readings. Parents were also questioned regarding passive smoking and any family history of asthma [20]. The relationship between RSV IgE titer and subsequent wheezing was then examined using maximal, convalescent titers obtained approximately 1 month after the acute infection [19].

The investigators concluded that the RSV IgE titer was predictive of the risk for developing wheezing from all causes later in life up to age 4 years. Only three out of 15 children who failed to develop RSV-IgE positive titers subsequently wheezed, as compared with seven out of 10 highly positive children, who had recurrent wheezing. Infants who fell in the middle of the spectrum in terms of RSV IgE titers also fell in the middle of the spectrum for wheezing [19]. By age 7 years, however, RSV IgE titers noted in infancy did not correlate with lung function or with the development of specific IgE antibodies to inhaled allergens [20].

The findings reported by Welliver *et al*. have proved difficult to repeat. Two additional studies [21,22] found no IgE antibodies to RSV in the nasopharyngeal secretions of populations of children hospitalized for RSV infection.

**Figure 1:**
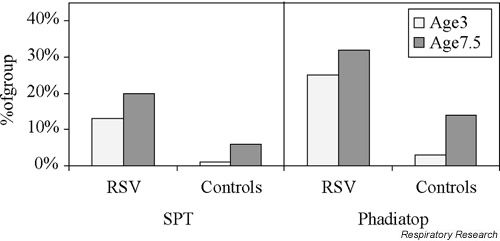
Allergic sensitization up to age 7.5 years in 44 children with respiratory syncytial virus (RSV) bronchiolitis in infancy and 89 control children. Phadiatop^®^ (Pharmacia Diagnostics, Uppsala, Sweden) is a screening test for serum IgE antibodies to inhaled allergens. SPT, skin prick test. Reproduced with permission from Sigurs *et al.*[14].

It is currently unclear what the relationship between RSV bronchiolitis and allergy may be in children who had been hospitalized for their infections. One group [10,11] found an increased risk for allergic sensitization up to age 6 years but could not verify it in the subset of children tested at age 10 years. Other studies [5,6,9] found no relationship between RSV bronchiolitis and allergic sensitization. However, the correlation between RSV bronchiolitis and allergy is supported by the previously cited studies by Sigurs and coworkers [4,14]. In those studies, skin prick tests and serum IgE tests were performed at ages 3 and 7.5 years for common food and inhalant allergens. At follow up at age 7.5 years, these tests were performed in 44 children who had been hospitalized for RSV LRTI and 89 control children. Allergic sensitization was significantly higher (*P* = 0.028) in the RSV group, with 18 out of 44 index cases (41%) versus 19 out of 86 control children (22%) having positive skin prick and serum IgE tests [14]. Fig. 1 shows allergic sensitization to inhaled allergens at ages 3 and 7.5 years.

### Studies of children with mild RSV infection

Children from the Tucson Children's Respiratory Study [17] who had suffered a mild RSV infection, and the control group in the study by Sigurs *et al*. [14] who had had subclinical RSV infection (as confirmed by the presence of IgG antibodies) were evaluated for atopy using skin prick tests and tests for specific serum IgE antibodies. No link was found between mild RSV infection and allergic sensitization at any age. Forster *et al*. [16] evaluated 609 children, of whom 44.7% had an elevated RSV IgG titer at age 1 year. They found a slightly increased risk for sensitization during the first year of life but not later in the RSV-infected children.

The severity of RSV infection or genetic or environmental factors could account for the differences observed in the study populations. It is clear, however, that the evidence for a connection between RSV infection and allergic sensitization is not conclusive, and further prospective studies are needed.

## Possible predisposing factors for bronchiolitis and postbronchiolitis symptoms

Although it seems clear that an association exists between RSV bronchiolitis and RAD, there may be predisposing factors that place a child at risk for both bronchiolitis and asthma. A prospective study of children enrolled in the Tucson Children's Respiratory Study [23] examined factors that affect wheezing before age 3 years and their relation to wheezing at age 6 years. Children under 1 year of age with respiratory illnesses who wheezed had evidence of diminished lung function even before exposure to RSV or other respiratory viral agents. It was hypothesized that these infants may have smaller airways that predispose them to wheezing, and that bronchial obstructive symptoms may have a variety of causes earlier in life. Maternal smoking appeared to be the strongest risk factor for wheezing, and may have been the causative agent for smaller lung development.

A prospective, longitudinal study of lung function, airway responsiveness, and LRTI [24] enrolled 253 infants up to age 2 years. There was a trend toward pre-existing reduced lung function in the 17 children who developed bronchiolitis (two out of 17 with a verified RSV infection). A follow-up study [25] later re-evaluated 120 members of this group at age 6 years. There was an increased incidence of asthma in children who had had bronchiolitis (44% versus 18% in control children) [24]. Airway responsiveness to inhaled histamine at a young age was correlated to asthma, poorer performance on spirometry testing, and lower respiratory tract symptoms in later childhood [25]. Those investigators argued that some genetic or environment factors (e.g. *in utero* exposure to maternal smoking) might have caused the initial predisposition to RAD, and that viral infections and other environmental insults may have inflicted further damage.

A multivariate analysis in the study by Sigurs *et al*. [14] evaluated several possible risk factors for asthma, including hereditary predisposition for atopy or asthma, male sex, exposure to second-hand smoke, and presence of indoor furred animals. In that study, RSV bronchiolitis was found to be the most significant risk factor for RAD (odds ratio 12.7; *P* < 0.001).

## Conclusion

The results of available retrospective and prospective studies (Table 1) of children with mild to moderate or severe RSV LRTI show that RSV infection in infancy is associated with an increased risk for bronchial obstructive symptoms many years after the infection and that lung function may be affected for several years. The mechanisms that underlie the connection are not clear, however, and it is disputed whether some predisposing factor is necessary for both the development of bronchiolitis and the subsequent wheezing, or whether the virus itself may cause postbronchiolitic symptoms. It is also possible that some predisposing factor(s) and the virus both contribute to the RAD [26]. One such predisposing factor could be a pre-existing diminished lung function before a respiratory virus is encountered. However, according to available studies, it appears certain that a family history of atopy and/or asthma does not explain the RAD symptoms that occur following RSV LRTI.

The connection between RSV LRTI and allergic sensitization is not as clear as the link between RSV and RAD, because the results of available studies differ (Table 2). The relationship between RSV and RAD suggests that preventive measures may be useful in reducing risk for asthma. Long-term, prospective, and randomized studies are needed to clarify whether prevention of severe RSV disease reduces the subsequent development of asthma and allergy. Use of currently available prophylactic agents such as palivizumab might offer a way to study the relationship between severe RSV infection and subsequent asthma.

## Abbreviations

FEV_0.75/1.0_ = forced expiratory volume at 0.75 s/1.0 s; LRTI = lower respiratory tract infection; PEFR = peak expiratory flow rate; RAD = reactive airway disease; RSV = respiratory syncytial virus.

## Figures and Tables

**Table 1:** Studies investigating the link between respiratory syncytial virus bronchiolitis and reactive airway disease

Reference	Year	Number of children (index/control)	Hospitalized	Age at follow up (years)	Presence of wheezing/asthma (index versus control)	*P* (odds ratio)	Abnormal PFTs in index cases
[5]	1978	35/35	Yes	8	51% versus 3%^*^	<0.0005	Yes
[6]	1982	130/111	Yes	10	42% versus 19%^*^	<0.001	Yes
[8]	1984	100/200	Yes	7	N/A	N/A	Yes
[15]	1984	59/177	No	8	44% versus 14%^†^	<0.0001	-
[9]	1987	51/24	Yes	2	60% versus 4%^†^	<0.01	-
[10]	1992	73/73	Yes	6	43% versus 15%^†^	<0.001	Yes
[12]	1993	70/70	Yes	2	44% versus 13%^†^	= 0.001	-
[4]	1995	47/93	Yes	3	23% versus 1%^†^	<0.001	-
[11]	1997	61/47	Yes	9–10	34% versus 13%^†^	(3.59)	Yes
[17]	1999	N/A	No	6	N/A	(4.3)	-
		N/A	No	13	N/A	(1.4; NS)	-
[13]	1999	105/105 and 102	Yes	3	N/A	NS at age 3 years	-
[14]	2000	47/93	Yes	7.5	23% versus 2%^†^	<0.001	-

All numbers were rounded to one significant figure. ^*^Cumulative rates of wheezing/asthma. ^†^Rates in the current year. N/A, no percentages or figures given; NS, not significant; PFT, pulmonary function test.

**Table 2:** Studies of the relationship between respiratory syncytial virus and indicators of allergic sensitization

Reference	Year	Number of children (index/control)	Age at follow up (years)	SPT performed	Serum IgE antibody obtained	Relationship as indicated by positive SPT and/or IgE
[6]	1982	130/111	10	Yes	No	No
[9]	1987	51/24	2	Yes	Yes	No
[10]	1992	73/73	6	Yes	No	Yes
[4]	1995	47/93	3	Yes	Yes	Yes
[14]	2000	47/93	7.5	Yes	Yes	Yes
[16]	1996	272/337	2	No	Yes	Yes
[11]	1997	61/47	9–10	Yes	No	No
[17]	1999	145/445	11	Yes	No	No

The studies evaluated indicators of allergic sensitization to common inhaled allergens, skin prick tests (SPT), and/or serum IgE antibodies.

## References

[B1] World Health Organization (2000). Bronchial Asthma WHO Fact Sheet No 206;.

[B2] Centers for Disease Control and Prevention (2000). Respiratory syncytial virus activity: United States 1999–2000 season.. MMWR Morb Mortal Wkly Rep.

[B3] National Center for Infectious Diseases, Respiratory and Enteric Viruses Branch.. http://www.cdc.gov/ncidod/dvrd/revb/.

[B4] Sigurs N, Bjarnason R, Sigurbergsson F, Kjellman B, Bjorksten B (1995). Asthma and immunoglobulin E antibodies after respiratory syncytial virus bronchiolitis: a prospective cohort study with matched controls.. Pediatrics.

[B5] Sims DG, Downham MA, Gardner PS, Webb JK, Weightman D (1978). Study of 8-year-old children with a history of respiratory syncytial virus bronchiolitis in infancy.. BMJ.

[B6] Pullan CR, Hey EN (1982). Wheezing, asthma, and pulmonary dysfunction 10 years after infection with respiratory syncytial virus.. BMJ.

[B7] Mok JY, Simpson H (1982). Outcome of acute respiratory tract infection in infants: preliminary report of seven-year follow-up study.. Br Med J Clin Res Ed.

[B8] Mok JY, Simpson H (1984). Symptoms, atopy, and bronchial reactivity after lower respiratory infection in infancy.. Arch Dis Child.

[B9] Carlsen KH, Larsen S, Bjerve O, Leegaard J (1987). Acute bronchiolitis: predisposing factors and characterization of infants at risk.. Pediatr Pulmonol.

[B10] Murray M, Webb MS, O'Callaghan C, Swarbrick AS, Milner AD (1992). Respiratory status and allergy after bronchiolitis.. Arch Dis Child.

[B11] Noble V, Murray M, Webb MS, Alexander J, Swarbrick AS, Milner AD (1997). Respiratory status and allergy nine to 10 years after acute bronchiolitis.. Arch Dis Child.

[B12] Osundwa VM, Dawod ST, Eblayal M (1993). Recurrent wheezing in children with respiratory syncytial virus (RSV) bronchiolitis in Qatar.. Eur J Pediatr.

[B13] Weber MW, Milligan P, Giadom B, Pate MA, Kwara A, Sadiq AD, Chanayireh M, Wittle H, Greenwood BM, Mulholland K (1999). Respiratory illness after severe respiratory syncytial virus disease in infancy in the Gambia.. J Pediatr.

[B14] Sigurs N, Bjarnason R, Sigurbergsson F, Kjellman (2000). Respiratory syncytial virus bronchiolitis in infancy is an important risk factor for asthma and allergy at age 7.. Am J Respir Crit Care Med.

[B15] McConnochie KM, Roghmann KJ (1984). Bronchiolitis as a possible cause of wheezing in childhood: new evidence.. Pediatrics.

[B16] Forster J, Tacke U, Krebs H, Streckert HJ, Werchau H, Bergmann RL, Schulz J, Lau S, Wahn U (1996). Respiratory syncytial virus infection: its role in aeroallergen sensitization during the first two years of life.. Pediatr Allergy Immunol.

[B17] Stein RT, Sherrill D, Morgan WJ, Holberg CJ, Halonen M, Taussig LM, Wright AL, Martinez FD (1999). Respiratory syncytial virus in early life and risk of wheeze and allergy by age 13 years.. Lancet.

[B18] Vickerstaff Joneja JM, Bielory L (1990). Understanding Allergy, Sensitivity, and Immunity: A Comprehensive Guide New Brunswick: Rutgers University Press;.

[B19] Welliver RC, Sun M, Rinaldo D, Ogra PL (1986). Predictive value of respiratory syncytial virus-specific IgE responses for recurrent wheezing following bronchiolitis.. J Pediatr.

[B20] Welliver RC, Duffy L (1993). The relationship of RSV-specific immunoglobulin E antibody responses in infancy, recurrent wheezing, and pulmonary function age 7–8 years.. Pediatr Pulmonol.

[B21] Toms GL, Quinn R, Robinson JW (1996). Undetectable IgE responses after respiratory syncytial virus infection.. Arch Dis Child.

[B22] Alarcon A, Walsh EE, Carper HT, LaRussa JB, Evans BA, Rakes GP, Platts-Mills TA, Heymann PW (2001). Detection of IgA and IgG but not IgE antibody to respiratory syncytial virus in nasal washes and sera from infants with wheezing.. J Pediatr.

[B23] Martinez FD, Wright AL, Taussig LM, Holberg CJ, Halonen M, Morgan WF, the Group Health Medical Associates (1995). Asthma and wheezing in the first six years of life.. N Engl J Med.

[B24] Young S, O'Keeffe PT, Arnott J, Landau LI (1995). Lung function, airway responsiveness, and respiratory symptoms before and after bronchiolitis.. Arch Dis Child.

[B25] Palmer LJ, Rye PJ, Gibson NA, Burton PR, Landau LI, Lesouef PN (2001). Airway responsiveness in early infancy predicts asthma, lung function, and respiratory symptoms by school age.. Am J Respir Crit Care Med.

[B26] Long CE, McBride JT, Hall CB (1995). Sequelae of respiratory syncytial virus infection: a role for intervention studies.. Am J Respir Crit Care Med.

